# Comparative Analysis of Procalcitonin and C-Reactive Protein in Bloodstream Infections Among Febrile Neutropenic Pediatric Cancer Patients

**DOI:** 10.7759/cureus.101495

**Published:** 2026-01-13

**Authors:** Tanya Pandey, RajKumar Kalyan, Tanya Sachan, Riya Singh, Sushmita Verma

**Affiliations:** 1 Microbiology, King George's Medical University, Lucknow, IND

**Keywords:** bloodstream infections, c-reactive protein, febrile neutropenia, pediatric cancer, procalcitonin, sepsis biomarkers

## Abstract

Febrile neutropenia is a serious complication in pediatric cancer patients receiving chemotherapy, often leading to bloodstream infections, where early diagnosis is critical. This prospective observational study, conducted at a tertiary care hospital in Northern India from August 2023 to July 2024, included 106 pediatric patients with chemotherapy-induced febrile neutropenia to compare the diagnostic performance of procalcitonin (PCT) and C-reactive protein (CRP). Bloodstream infections were confirmed in 32 patients (30.2%), comprising 20 Gram-negative, 11 Gram-positive, and one fungal isolate. PCT levels were significantly higher in Gram-negative infections (19.27 ± 10.56 ng/mL) than in Gram-positive infections (2.57 ± 2.35 ng/mL, p<0.01), while CRP showed less specificity (150.0 ± 35.2 mg/L vs. 45.0 ± 20.8 mg/L, p<0.05). PCT demonstrated superior diagnostic accuracy (sensitivity 89.2%, specificity 83.5%, AUC 0.93) compared to CRP (sensitivity 76.3%, specificity 62.7%, AUC 0.76). Elevated PCT levels correlated with higher intensive care admissions, prolonged hospital stays, and delayed antibiotic de-escalation (p<0.001). These findings indicate that PCT is a more reliable biomarker than CRP for early detection of bloodstream infections in febrile neutropenic pediatric cancer patients, particularly for Gram-negative bacteremia, and its routine incorporation may enhance infection management and clinical outcomes in pediatric oncology.

## Introduction

Febrile neutropenia (FN) is a critical oncological emergency and a serious complication frequently resulting from chemotherapy. In immunocompromised patients, fever may be the only indication of an underlying infection, necessitating prompt medical evaluation and treatment [1]. Neutropenia significantly alters the host’s inflammatory response, prompting the exploration of various biomarkers for the detection of infections. Several cytokines have been investigated to identify reliable markers for stratifying infectious risk in children with FN, but their role remains under evaluation. The most extensively studied biomarkers include procalcitonin (PCT), C-reactive protein (CRP), and interleukins (IL-6, IL-8, and IL-10), all of which are involved in inflammatory pathways [2-5].

Procalcitonin, a precursor of the hormone calcitonin, is produced by various organs in response to bacterial infection [6]. It demonstrates greater specificity for bacterial infections than other acute-phase reactants and is synthesized independently of leukocyte count or function [7]. Several PCT assays have been approved by the US FDA for prognostication and guiding antimicrobial therapy; however, their clinical utility in children remains debated [8]. Studies have shown that PCT is superior to CRP in distinguishing bacterial infections during the first day of febrile neutropenia and may also predict FN outcomes [9].

Despite these findings, evidence comparing the diagnostic accuracy of PCT and CRP in pediatric patients with chemotherapy-induced FN remains limited and inconclusive. This gap underscores the need for systematic evaluation of these biomarkers in this high-risk population. Therefore, the present study aimed to compare the diagnostic value of PCT and CRP in the early identification and differentiation of bloodstream infections in pediatric cancer patients with febrile neutropenia, with the goal of supporting timely and appropriate treatment decisions.

## Materials and methods

Study design and setting

This prospective, hospital-based observational study was conducted in the Department of Microbiology in collaboration with the Department of Pediatrics at Gandhi Memorial and Associated Hospitals, King George’s Medical University (KGMU), Lucknow, Uttar Pradesh, India, a large ~4,500-bed tertiary care teaching hospital, from August 2023 to July 2024.

Sample size calculation

The sample size was calculated using the formula

$n = \frac{Z^{2} P (1 - P)}{D^{2}}$

as described by Radhakrishnan et al. [10]

where Z = 1.96 corresponds to a 95% confidence level. The prevalence (P) was taken as 7.5%, representing the reported prevalence of bloodstream infections among pediatric patients with febrile neutropenia based on previously published literature. The margin of error (D) was set at 5%, which is conventionally accepted in hospital-based observational studies to ensure adequate precision while maintaining feasibility. Based on these parameters, the minimum required sample size was calculated to be 106 patients.

Study population

All children under 18 years of age with a confirmed diagnosis of hematological or oncological malignancy who developed febrile neutropenia during treatment were eligible for enrollment. Febrile neutropenia was defined as an absolute neutrophil count (ANC) below 500 cells/µL, or an ANC between 500-1000 cells/µL with an anticipated decline to below 500 cells/µL within 48 hours, accompanied by either a single oral temperature of ≥ 38.3 °C, a sustained temperature of ≥ 38.0 °C for more than one hour, or two febrile episodes within 24 hours [11]. Patients who had received antibiotic therapy prior to admission or whose parents or guardians did not provide informed consent were excluded. Additionally, participants initially enrolled but later found to have incomplete clinical or laboratory data, protocol deviations, or withdrawal of consent were eliminated from the final analysis.

Data and sample collection

Demographic, clinical, and laboratory information were recorded using a structured proforma. At the onset of each febrile episode, blood samples were collected for the following investigations:

Blood Culture

Two venous blood samples were collected aseptically and inoculated into BacT/ALERT® culture bottles (bioMérieux, Marcy-l’Étoile, France) in recommended volumes and incubated in the BacT/ALERT® automated blood culture system (bioMérieux, Marcy-l’Étoile, France). Growth in at least one bottle was considered positive with clinical sign of infection, but for organisms with a high likelihood of contamination, including coagulase-negative *Staphylococci (CoNS), Bacillus *spp*., *and *Corynebacterium *spp., true bacteremia was confirmed if there was supporting clinical evidence of infection and the same organism was isolated in a subsequent blood culture, since these organisms frequently represent contamination. Positive cultures were subcultured on blood agar, chocolate agar, and MacConkey agar, and isolates were identified using MALDI-TOF MS (bioMérieux, Marcy-l’Étoile, France).

Serum PCT levels were measured using the Mini VIDAS® automated immunoassay system (bioMérieux, Marcy-l’Étoile, France) according to the manufacturer’s instructions.

CRP was measured using a slide agglutination CRP latex kit (LABKIT, CHEMELEX S.A., Barcelona, Spain) according to the manufacturer’s instructions.

Study objectives

The primary objective was to compare the diagnostic performance of PCT and CRP in detecting bloodstream infections in febrile neutropenic pediatric cancer patients. Secondary analyses included evaluating their correlation with Gram-negative versus Gram-positive bacteremia and associated clinical outcomes.

Statistical analysis

Data were analyzed using IBM SPSS Statistics for Windows, version 26.0 (IBM Corp., Armonk, NY). Continuous variables were expressed as mean ± standard deviation (SD) and compared using the Student’s t-test. Categorical variables were analyzed using the Chi-square or Fisher’s exact test, as appropriate. Diagnostic accuracy was evaluated using sensitivity, specificity, and receiver operating characteristic (ROC) curve analysis. The area under the ROC curve (AUC) with 95% confidence intervals was calculated to assess overall discriminative ability. Optimal cut-off values were determined using the Youden index, assuming microbiologically confirmed bloodstream infection as the reference standard. A p-value less than 0.05 was considered statistically significant.

Ethical approval

The study was approved by the Institutional Ethics Committee of King George’s Medical University, Lucknow (IRB Approval No: XIV-PGTSC-IIA/P71). Written informed consent was obtained from parents or legal guardians of all participants.

## Results

Demographic profile

The study included 106 pediatric patients aged 2-15 years, with a mean age of 6.42 ± 2.68 years. Half of the participants were between 5-10 years (53 patients, 50.0%), followed by 39 patients (36.8%) in the 2-4-year age group and 14 patients (13.2%) in the 11-15-year group. The cohort showed a male predominance, with 85 males (80.2%) and 21 females (19.8%). Among the 106 participants, bloodstream infections (BSIs) were confirmed in 32 children (30.2%) based on positive blood culture results. Gram-negative organisms were the predominant pathogens, isolated in 20 cases (62.5%), followed by Gram-positive organisms in 11 cases (34.4%). Fungal growth was detected in 1 case (3.1%) (Table 1).

**Table 1:** Baseline demographic characteristics of the study cohort (N=106).

Variable	Category	N	Percentage (%)
Age Group	2-4 years	39	36.8
	5-10 years	53	50.0
	11-15 years	14	13.2
Average Age	Mean ± SD	6.42 ± 2.68	-
Sex Distribution	Male	85	80.2
	Female	21	19.8
Blood Culture Results	Positive	32	30.2
	Negative	74	69.8

PCT and CRP levels

PCT levels were significantly higher in Gram-negative cases (20/32; 62.5%) at 19.27 ± 10.56 ng/mL, compared to Gram-positive cases (11/32; 34.4%) at 2.57 ± 2.35 ng/mL (p< 0.01). CRP levels were also higher in Gram-negative infections (150.0 ± 35.2 mg/L) than in Gram-positive infections (45.0 ± 20.8 mg/L) (p < 0.05) (Table 2).

**Table 2:** Comparison of procalcitonin (PCT) and C-reactive protein (CRP) levels in bloodstream infections caused by Gram-negative and Gram-positive bacteria. Values are presented as mean ± standard deviation. PCT and CRP levels were significantly higher in Gram-negative infections compared to Gram-positive infections (p< 0.05 considered significant).

Parameters	Gram-negative infections	Gram-positive infections	p-value
Mean PCT Level (ng/mL)	19.27 ± 10.56	2.57 ± 2.35	< 0.01
Mean CRP Level (mg/L)	150.0 ± 35.2	45.0 ± 20.8	< 0.05

Diagnostic accuracy

**Figure 1:**
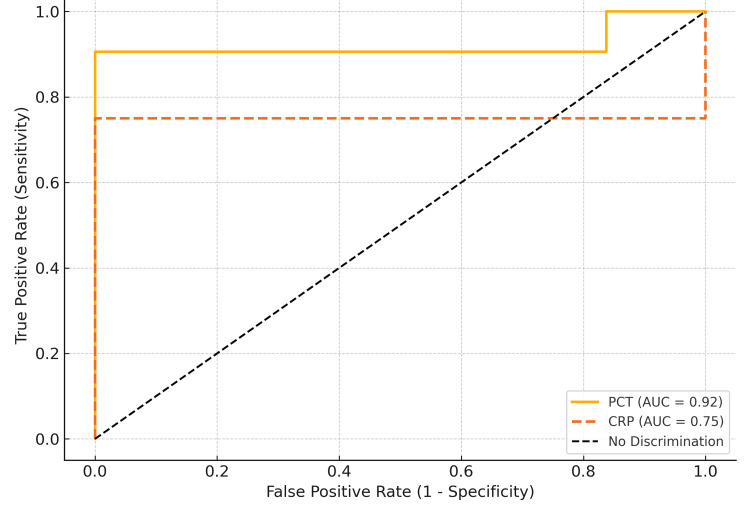
Receiver operating characteristic (ROC) curves showing diagnostic performance of PCT (AUC=0.93) and CRP (AUC=0.76) for bloodstream infections.

Using blood culture as the reference standard, PCT demonstrated higher diagnostic accuracy than CRP. PCT showed a sensitivity of 89.2%, specificity 83.5%, positive predictive value (PPV) 70.7%, and negative predictive value (NPV) 95.4%. In contrast, CRP had a sensitivity of 76.3%, specificity 62.7%, PPV 46.2%, and NPV 85.2%. Receiver Operating Characteristic (ROC) analysis yielded an AUC of 0.93 for PCT and 0.76 for CRP, confirming the superior diagnostic performance of PCT (Table 3, Figure 1).

**Table 3:** Diagnostic accuracy of PCT and CRP in detecting bloodstream infections, using blood culture as the reference standard.

Parameters	PCT (%)	CRP (%)
Sensitivity	89.2	76.3
Specificity	83.5	62.7
Positive Predictive Value (PPV)	70.7	46.2
Negative Predictive Value (NPV)	95.4	85.2

Serial biomarker trends

Serial measurements revealed dynamic trends for both biomarkers. PCT levels rose from 15.3 ± 8.4 ng/mL at admission to a peak of 19.2 ± 10.5 ng/mL at 48 hours, then declined to 12.5 ± 6.3 ng/mL by 72 hours. CRP levels showed a similar pattern, peaking at 150.0 ± 35.2 mg/L at 48 hours, but declined more gradually (Table 4).

**Table 4:** Serial changes in PCT and CRP levels at admission, 24, 48, and 72 hours in febrile neutropenic pediatric cancer patients with bloodstream infections.

Time Interval	Mean PCT Level (ng/mL)	Mean CRP Level (mg/L)
At Admission	15.3 ± 8.4	130.5 ± 30.6
24 Hours	17.8 ± 9.1	140.2 ± 33.7
48 Hours	19.2 ± 10.5	150.0 ± 35.2
72 Hours	12.5 ± 6.3	120.8 ± 28.4

Correlation with treatment and outcomes

Patients were stratified based on admission PCT levels into high (> 0.5 ng/mL) and low (< 0.5 ng/mL) groups. Empirical antibiotic therapy was initiated in 100.0% of high-PCT patients compared to 60.8% of low-PCT patients (p< 0.001). Antibiotic de-escalation by Day 5 occurred in 72.0% of low-PCT patients versus 18.0% of high-PCT patients (p< 0.001).

The mean duration of antibiotic therapy and hospital stay was significantly longer in the high-PCT group (10.2 ± 2.3 days and 12.7 days, respectively) compared with the low-PCT group (6.1 ± 1.8 days and 8.4 days, respectively; p< 0.001 and p=0.004). ICU admission was required in 54.0% of high-PCT patients compared with 18.0% of low-PCT patients (p< 0.001) (Table 5).

**Table 5:** Clinical outcomes and impact of PCT levels on antibiotic use and hospital course. Patients were stratified into high (> 0.5 ng/mL) and low (< 0.5 ng/mL) PCT groups.

Clinical Parameters	High PCT (> 0.5 ng/mL) (n = 32)	Low PCT (< 0.5 ng/mL) (n = 74)	p-value
Empirical antibiotic initiation (%)	100.0% (32/32)	60.8% (45/74)	< 0.001
De-escalation of antibiotics by Day 5 (%)	18.8% (6/32)	71.6% (53/74)	< 0.001
Mean duration of antibiotic therapy (days)	10.2 ± 2.3	6.1 ± 1.8	< 0.001
Average hospital stay (days)	12.7	8.4	0.004
ICU admission required (%)	53.1% (17/32)	17.6% (13/74)	< 0.001

## Discussion

Diagnostic superiority of procalcitonin over C-reactive protein

This study demonstrates that PCT has superior diagnostic performance compared to CRP in detecting bloodstream infections among febrile neutropenic pediatric cancer patients. PCT exhibited higher sensitivity (89.2%) than CRP (76.3%), particularly for Gram-negative bacteremia, making it a reliable early biomarker for bacterial infections.

These findings align with Beqja-Lika et al. [12], who reported that PCT had higher sensitivity (97.4%) and specificity (96.6%) in distinguishing sepsis from systemic inflammatory response syndrome (SIRS), whereas CRP, despite high sensitivity (98.6%), had lower specificity (75%), limiting its value as a standalone diagnosticmarker. Similarly, Shilpakar et al. [13] observed significantly higher median PCT levels in bacteremic pediatric patients (3.25 ng/mL vs. 0.51 ng/mL, p< 0.01), whereas CRP levels did not differ significantly (119.3 mg/L vs. 94.5 mg/L, p=0.07).

Correlation with disease severity and diagnostic accuracy

PCT not only identified infections effectively but also correlated strongly with disease severity. Luzzani et al. [14] reported markedly elevated PCT levels in septic patients (3.65 ng/mL vs. 0.4 ng/mL, p< 0.0001), with superior diagnostic accuracy (AUC 0.925 vs. 0.677) and stronger correlation with disease severity (Spearman’s rho 0.73 vs. 0.41) compared to CRP. Massaro et al. [15] similarly found elevated PCT in severe infections (6.7 ng/mL vs. 0.6 ng/mL, p=0.0075) with higher sensitivity and specificity at a 0.245 ng/mL threshold.

Serial biomarker trends and monitoring utility

Serial measurements showed that PCT levels peaked at 48 hours (19.2 ± 10.5 ng/mL) and declined by 72 hours (12.5 ± 6.3 ng/mL), reflecting infection progression and response to therapy. In contrast, CRP remained elevated longer, peaking at 48 hours (150.0 ± 35.2 mg/L) and declining more slowly, reinforcing its role as a marker of inflammation rather than a dynamic infection indicator.

Meisner et al. [16] demonstrated similar findings in critically ill patients, showing that PCT levels correlated strongly with organ dysfunction scores, whereas CRP did not differentiate disease severity effectively. Secmeer et al. [17] further highlighted that PCT declined earlier in patients without infections, whereas CRP showed slower resolution, underscoring PCT’s responsiveness to treatment.

Doganci et al. [18] reported that persistent elevation of CRP, PCT, and leukocyte levels on day 3 was associated with increased 3-month mortality in adult ICU patients with pneumosepsis. In contrast, our study focused on early diagnosis in pediatric febrile neutropenic patients, showing that PCT was more accurate than CRP in detecting bloodstream infections, particularly Gram-negative sepsis. This emphasizes the complementary roles of these biomarkers in both diagnosis and prognosis, while also highlighting differences between pediatric and adult populations.

Clinical implications for antimicrobial stewardship

High PCT levels in our cohort were associated with positive blood cultures, prolonged antibiotic use, and longer hospital stays. Conversely, low PCT levels allowed earlier antibiotic de-escalation (by day 5 in 72% of cases), shorter therapy duration, and reduced hospital stay, emphasizing the utility of PCT in guiding rational antimicrobial therapy in febrile neutropenic pediatric patients. Liu et al. [19] similarly reported that combined PCT and CRP measurement improved diagnostic accuracy and informed clinical management.

Comparative diagnostic roles

While CRP remains a useful marker for general inflammation, our study shows that PCT is more clinically informative in febrile neutropenic pediatric cancer patients, particularly for early detection of Gram-negative bloodstream infections and guiding antibiotic decisions. Cho et al. [20] noted higher CRP AUC for infection detection in hematologic patients, but PCT had a stronger association with clinical outcomes, consistent with our findings.

Study limitations

This study was conducted at a single tertiary care center, which may limit the generalizability of findings. Additionally, the sample size for Gram-positive and fungal infections was small, potentially affecting subgroup analyses. Future multicenter studies with larger cohorts are needed to validate these results.

## Conclusions

PCT demonstrated superior diagnostic accuracy and clinical utility compared to CRP in detecting bloodstream infections among febrile neutropenic pediatric cancer patients. PCT not only showed higher sensitivity for early infection detection but also provided better correlation with infection severity and treatment response, particularly in cases of suspected bacterial bloodstream infections and Gram-negative bacteremia. Its dynamic profile makes it a more effective biomarker for guiding early clinical decisions, including risk stratification and antimicrobial escalation or de-escalation, and optimizing antimicrobial therapy in clinically severe presentations. Incorporating PCT measurement into standard diagnostic protocols for febrile neutropenia as an adjunct to clinical assessment could enhance infection management and reduce morbidity in this high-risk population.
